# Attitudes of Peer Support Workers towards the Medical Model: A Qualitative Study from the Viewpoints of Peer Support Workers and Mental Health Staff

**DOI:** 10.1007/s10597-025-01454-z

**Published:** 2025-02-13

**Authors:** Guillermo Ruiz-Pérez, Sebastian von Peter

**Affiliations:** Department of Psychiatry and Psychotherapy, Brandenburg Medical School, Immanuel Klinik Rüdersdorf, Rüdersdorf, Germany

**Keywords:** Peer support work, Medical model, Attitude, Recovery, Implementation

## Abstract

Attitudes of Peer Support Workers are generally expected to be grounded in recovery philosophy. They may encounter resistance from Mental Health Workers, which tend to be more closely aligned with the medical model. We aim to investigate how PSWs relate in clinical contexts to some main aspects of medical model-based attitudes. This research is part of the larger ImpPeer-Psy5 project, involving 57 interviews with PSWs, MHWs, and service users. The findings presented here were derived from a qualitative sub-study with 5 more interviews and one focus group with MHWs. Some PSWs view psychiatric diagnoses as helpful for their own recovery and interactions with clients, while others find them reductive and stigmatizing. Similarly, PSWs hold varying opinions on psychiatric drugs, with some seeing them as beneficial and necessary, while others have critical views. MHWs' expectations on PSWs may not always align with their attitudes.

**Supplementary Information:**

The online version contains supplementary material available at 10.1007/s10597-025-01454-z.

## Introduction

Discussions and analyses of the role of the medical model in mental healthcare have engaged both empirical and theoretical concerns in recent years (Barnes et al., 2022; Carter & Hall, 2017; Hogan, 2019; Huda, 2019; Maiese, 2021). In the following, we refer to the ‘medical model’ in a similar register as many of its critics, referencing the perception of those who consider more recent iterations (for instance the bio-psycho-social model) as superficial updates of the old biologism simply rendered in modern terminology (Read et al., 2009). These critics describe the “medical model” as asserting the existence of an essential biological dysfunction, at times influenced by concomitant conditions (psycho-social), the latter targeted by corresponding therapies (Joyce, 1980). In this line of thinking, mental healthcare practices involve medically justified diagnostic labels to identify groups of symptoms or underlying pathologies, followed by the application of corresponding treatment strategies (Zachar & Kendler, 2007).This logic, which has an eminently pragmatic character, fits within the biomedical framework of causality, leaving it mostly without criticism (Walter, 2013). Howver. many authors and scholars have emphasized the eminent social and interactive aspects of psychiatric conditions, which they argue must be seriously considered (Barker, 2008; Farre & Rapley, 2017; Hogan, 2019).

Over the last few decades, the medical model has been criticized from various perspectives, some of which advocate for more comprehensive theoretical frameworks such as “enactivism” or more socio-ecological conceptualizations. These approaches underpin such models as the Open Dialogue approach, for example (Galbusera & Kyselo, 2018). These frameworks transcend the dominance of either a purely biological or social focus, instead embracing more integrated, socially interactive, or humanistic models of assessment and support (Maiese, 2021; Seikkula, 2021). The “relational” model, challenging both the objectivism and individualism of the medical model, is another example (Jurgens, 2023). The recovery model is another important example, although somewhat more elusive in its tenets (Ellison et al., 2016), which aims to contribute to a more integrated and complex understanding of mental health conditions (Davidson et al., 2010; Meehan et al., 2008) by recombining principles such as connectedness, empowerment, personal responsibility, hope, and identity (Leamy et al., 2011). The central critique of the recovery model directed at the medical model concerns the latter’s reductionism in medicalizing psychological crises, along with the individualism that stems from it. As an alternative, the recovery model focuses on a more positive outlook towards overcoming crises, moving away from the deficit-oriented and often hopeless attitude that usually underlies biological approaches (Cruwys et al., 2020).

Central to the recovery approach are various forms of peer support (Slade et al., 2014; Wallcraft et al., 2011; Wright et al., 2016), either outside of the mental healthcare system, such as autonomous, self-organized, mutual peer support groups (Penney, 2018), or more formalized positions within this system, such as Peer Support Workers (PSWs). PSWs who have experienced mental health crises and discrimination themselves tend to promote less pathologizing and medicalizing perspectives regarding the mental health of patients and clients (Frieh, 2024; Moore & Zeeman, 2020). Even though their attitudes towards the medical model are not homogeneous (Adams et al., 2023; Moran et al., 2013), the capacity for a more nuanced, less medically based approach to mental health care is broadly attributed to PSWs and their work, even by themselves (Frieh, 2024). The discussion section elaborates on this point in greater detail. At the same time, PSWs only have the possibility to action these perspectives if they are effectively integrated into mental healthcare teams. This tension surfaces the dilemma of how to implement PSW in a healthcare system that is widely recognized as being primarily medically dominated by both critics (Barnes et al., 2022; Moncrieff, 2022; Read et al., 2009) and advocates.

Building on this question, our study aims to provide further insight into how PSWs in professional positions relate to the medical model, both from their own perspectives and the perspectives of the MHWs who collaborate with them. Our primary research questions are: (1) What are the attitudes of PSWs towards key aspects of the medical model, such as the use of diagnostic categories and psychotropic drugs? (2) How do these attitudes interact with MHWs’ expectations of PSWs? and (3) What impact does this interaction have on the implementation of PSW in clinical contexts? The overarching aim is to identify potentially conflicting or enabling factors that may facilitate or obstruct the implementation of PSW in mental health care services.

## Methods

### Study Context

Our research is part of a larger study known as ImpPeer-Psy5, which focuses on the implementation of peer support work within the German mental health care system. The implementation of PSW in Germany varies significantly across different regions and institutions, and is influenced by a diverse range of local, regional, and federal regulations and conditions. The public mental healthcare system in Germany depends largely on psychiatric hospitals, with approximately 600 hospitals spread throughout the country. These hospitals primarily provide inpatient services, followed by a smaller proportion of day clinics, outpatient services, and a very small percentage of home treatment services. The services offered by psychiatrists and psychologists/psychotherapists are regulated by the Fifth German Social Code Book (SGB V) and financed by contributions from approximately 90 statutory health insurance companies. Approximately 70% of all expenditure allocated to individuals with mental health diagnoses comes from this sector, which is known as the ‘mental healthcare sector’ to distinguish it from the ‘psychosocial sector’. The latter category encompasses a diverse range of vocational, residential, and psychosocial support services, which are overseen by Social Code Books IX and XII and funded by pension funds and individual social benefits. Although there are no exact data, it is believed that the majority of the roughly 2500 PSWs in Germany are employed in this latter sector (SGB IX and XII), which raises critical questions regarding the categorization of PSW employment, the services they provide, and how remuneration is imagined and allocated by the state. To this end, this study specifically examined the incorporation of PSWs in the mental healthcare sector, with a primary focus on those working in psychiatric hospital departments under the jurisdiction of SGB V.

### Study Design

The methodological design and research processes of the ImpPeer-Psy5 study have been comprehensively detailed in a recent publication (von Peter et al., 2024), thus they will be described only briefly here. To allow for more comprehensive and valid results, this study used a mixed-method QUAL1-QUAN-QUAL2 design. The two qualitative phases of the study (QUAL1 and QUAL2) were conducted at Brandenburg Medical School (MHB), where this sub-study has also been carried out. An ethical approval was secured for the Impeer-Psy5 study at Brandenburg Medical School (No. E-01-20200826).

For the QUAL1 phase, a collaborative research approach was employed, which involved the cooperation of researchers with and without lived experience with psychiatric care (von Peter, 2017). To delve more deeply into the results of the QUAL1 (and QUAL2) phase, a range of sub-studies were conducted during the QUAL2 phase, one of which examined the attitudes of PSWs and MHWs towards the medical model after the introduction of PSW programs. This study, presented here, incorporates information from the QUAL1 phase and additional data obtained during the QUAL2 phase. Although this sub-study was largely completed by one of the authors, it received valuable input from other ImpPeer-Psy5 team members, as the QUAL1 phase was conducted in a collaborative manner, and the results of the QUAL2 evaluations were shared amongst the entire research team.

### Recruitment

The participants for both qualitative phases of the study were selected from three main stakeholder groups: (1) PSWs currently or previously employed in mental healthcare settings, such as psychiatric hospital units or integrated care teams; (2) users who have received PSW; and (3) MHWs who work alongside PSWs, including those trained in psychiatry, psychology, social work, and nursing. PSWs in both qualitative phases were recruited through the German peer support institution EX-IN (Experience-Involved) and with help from other peer support organizations (including EUTB, Lebensart Münster, and UPSIDES). Information about the study was disseminated on social media platforms dedicated to peer support-related topics in order to increase the pool of potential participants. A total of 57 individuals participated in the QUAL1 phase, including 32 PSWs, 19 MHWs, and six service users. In the QUAL1 phase, participants were selected using a combination of snowball sampling and purposive case selection with the aim of creating a balanced sample that represented all stakeholder groups, sociodemographic backgrounds, regions, and healthcare sectors.

In the QUAL2 phase, two recruitment strategies were used. For the interviews (n = 5), we used purposive sampling, aimed at MHWs who had practical experience of co-working with PSWs. We recruited MHWs who had already taken part in QUAL1 for the focus group (n = 3). This allowed us to ground the discussion of the focus group on the material gained from QUAL1, and to facilitate meta-reflections from the participants. For the sub-project presented here, we recruited additional MHWs in order to balance the sample with participating PSWs, who constituted the majority of the participants during QUAL1. Further sociodemographic information is included in the supplementary annex (Supplementary Material 1 — Sociodemographic data).

### Data Collection and Analysis

Detailed information about data collection and analysis can be found in a methodological publication (von Peter et al., 2024). The QUAL1 phase commenced on February 1, 2021, and concluded on July 30, 2021, followed by the QUAL2 phase, which started on September 1, 2022, and concluded on December 15, 2022. To promote a dialogical interview setting, the interviews were conducted in pairs, with each pair comprising two team members, one of whom had lived experience with psychiatric care, and another who did not. Online interviews were conducted during the COVID-19 pandemic period. The interviews were recorded, transcribed by a third-party service, and analyzed collaboratively by both individual team members and the entire research group in an iterative manner. Based on the central themes and questions that emerged during this QUAL1 phase, an interview guide was developed for the QUAL2 phase (Supplementary Material 2 — Interview Guide).

During the QUAL2 phase, in which this subproject was conducted, five additional interviews and one focus group were conducted in the context of this sub-study. GRP conducted the interviews alone. The focus group was moderated in tandem with a researcher with lived experience and trained in PSW. The interviews and focus group were conducted online, recorded, transcribed, and analyzed using a modified version of thematic analysis for collaborative research, which was carried out both inductively and deductively (Braun & Clarke, 2022). Initially, the QUAL2 material was analyzed inductively. The resulting codes were merged with codes and categories from the QUAL1 phase (Supplementary Material 3—Code system), and transcripts from both study phases were re-coded with this overarching code system. The data was managed and analyzed using MAXQDA software. A thematic analysis was performed by the entire research team in order to ensure trustworthiness and credibility: in various group sessions, the primary codes and subcodes were organized into thematic groups and categories. A more formalized check of inter-rater reliability was not possible because only one author (GRP) implemented recoding of the material in the context of his PhD research program.

During this sub-study, we operationalized two key aspects of the medical model: (1) the assignment of medical diagnoses to a group of symptoms, understood as emerging from a biological dysfunction and (2) the pharmacological intervention designated to treat it. We used this operationalization following Fuller’s (2017) definitions of both the ‘old’ and the ‘new medical model.’ From this standpoint, we developed a questionnaire for both the interviews and the focus group, trying to cover the variety of subrogated aspects in which the medical model may play a role in the everyday work of and with PSWs (Supplementary Material 2 — Interview Guide).

## Findings

The first part of the results section explores the diverse attitudes of PSWs towards the medical model. The second part demonstrates how PSWs intersect with MHWs and the expectations that the latter put on the former. The third part identifies conflicts that arise from the divergence between PSWs’ attitudes and MHWs’ expectations.

### The Diversity of the PSWs’ Attitudes

Several participants emphasized that the recovery principles, in which PSWs are usually trained, cannot be universally applied to every PSW. This makes it difficult to generalize the degree to which a PSW may actively embody a recovery orientation.

„Well, as I said, the group, I have already experienced many peers with us. The group was as heterogeneous, as people are. (MHW, Interview, QUAL1).

Participants stressed that each PSW possessed unique experiences with crises and the role of the mental health system or particular caregivers during these times. As a result, perspectives on the nature of mental health care and the approach to crises can differ significantly.

#### Attitudes on Psychiatric Diagnosis

For some PSWs, a medical diagnosis was not deemed crucial to their job, and they strived to refrain from using such terminology. Other PSWs acknowledged that a diagnosis could be beneficial during times of crisis or in their daily work with service users. One PSW noted that it was important for her to know which diagnoses service users had received in order to approach them more effectively.

“With peer support workers, yes, the idea is that you don’t go over diagnoses, but I find that sometimes that gets mixed up, too. Sometimes I need to know a diagnosis to be able to classify behavior.” (PSW, Interview, QUAL1)

One of the participating PSWs noted that other PSWs found diagnoses not only helpful for being able to work better, but also they were an important aspect of their own recovery processes. In these cases, diagnoses enabled them to define and describe the nature of their crises and how they could cope with them. A diagnosis can create a sense of belonging to a group with whom they can share their experiences.

“But I know people who say that ‘the diagnosis is good for me’, because they didn’t have one before. I have to think of one woman in particular who heard at some point about this diagnosis and that reassured her because she had the feeling that ‘you can be the way I am, that’s what’s described and there are other people who are like me, so I actually still belong to them.’” (PSW, Interview, QUAL1)

Other PSWs participating in our study perceived the diagnosis as potentially stigmatizing. It was argued that the attitudes of MHWs were usually shaped by diagnoses, leading to a deficit-oriented and labeling approach towards service users:

“I think - and I am firmly convinced of this - that in psychiatry itself the individual diagnoses are stigmatized differently, also by the professional staff. So, I think someone with a mild depression is quite a nice patient, but someone – let’s say - with a severe borderline personality disorder...” (PSW, Interview, QUAL 1)

As a result, some of the participating PSWs de-identified from their diagnostic ascriptions, instead advocating for a non-classificatory position. One PSW reported how her opinions on service users had been dismissed or generalized by other staff members, who strategically referred to her diagnosis.

“I expect not to be defined by my diagnosis, but to be seen as a human being, a person with a wealth of experience. (…) But then she [staff member] said - also with such a snippy tone: ‘I would like to know what kind of diagnosis you have that you can say something like that here’. And then I said, ‘Oh, sorry, that has nothing to do with my diagnosis.’” (PSW, Interview, QUAL 1)

#### Attitudes On Psychopharmacological Substances

In our study, the majority of PSWs emphasized the significance of allowing service users to independently determine their use of psychopharmacological medication. Additionally, participants reported that service users usually wanted to talk about their drug treatment with the PSW. Participant PSWs emphasized the importance of self-determination and self-responsibility in these issues.

“When I support people, they often ask me, do you take medication? Then I say no, I do not take it, but I am responsible for that, and that’s not funny for me sometimes. I sometimes sit on the floor and cry, but I am responsible for myself.” (PSW, Interview, QUAL 2)

During our interviews, many PSWs indicated that they did not feel competent in addressing medication-related questions. Some PSWs and MHWs reported the danger of PSWs being used as a way of indirectly convincing service users to take medication, because the latter may trust PSWs more than MHWs. In contrast, PSWs viewed themselves as neither responsible for convincing users to take medication nor for the opposite.

“But, of course, I don’t see it as my job to try to establish treatment compliance as far as the medications are concerned. And I think that’s often a balancing act” (PSW, Interview, QUAL 1)

Given the frequent interest of service users in relation to this topic, some of the participating PSWs described the challenge of an “in-between-role” between MHWs and users:

“The topic of medication is a very difficult topic in clinical contexts as a peer worker because you simply have this in-between role. Of course, I know about the side effects myself, and I know that people do not like to take them. But I take medication myself, and I feel that it does good or helps.” (PSW, Interview, QUAL 1)

PSWs usually try to resolve this tension by referring to medical doctors when asked about medication. They tried to be cautious and shy back from giving advice. Most PSWs interviewed tended to keep themselves out of these discussions on medications.

### MHWs’ Expectations on PSWs and How They Interact with Them

In Sect. "The Diversity of the PSWs’ Attitudes", it is evident that the attitudes and positions of PSWs cannot be generalized. This may be one reason why some MHWs repeatedly described PSWs’ attitudes as deviating from their original expectations. For example, they reported that some PSWs surprisingly held on to their diagnosis, which had been assigned while they were a patient, a decision that the MHWs had not expected.

“Excitingly, the colleague, the peer worker held on to the diagnoses for a long time. And that really surprised me because I thought she was somehow hungry to get rid of them and have nothing more to do with them. But she was able to justify it well for herself and said that it simply helped her a lot, that at that time, yes, somehow it had a name that she could relate to and that she understood herself better.” (MHW, interview, QUAL 1)

Furthermore, the fact that some PSWs talked positively about psychopharmacological drugs came as a surprise to some of the participating MHWs, who expected a more conservative stance among PSWs.

“I am not against medication, but I think that if someone says I want to try treatment without medication first, then an institution should also try that. And then I sometimes say to the peer support workers: ‘Bring in your experience’, and then they often say: ‘Well, it does need medication’” (MHW, interview, QUAL 1)

Although PSWs are usually expected to have a critical view of medication and diagnosis, MHWs are facing a more diverse landscape of attitudes. On the other hand, too much criticism from PSWs was reported to lead to conflicts, as described in the following section. In contrast to this position, one MHW of the focus group noted that it would be desirable if PSWs were more involved in decision-making in relation to drug treatments instead of keeping themselves away from this topic, as was reported in Sect. "MHWs’ Expectations on PSWs and How They Interact with Them".

### Conflicts Resulting From the Interaction between MHWs and PSWs

Some MHWs had expected PSWs to help deconstruct the mental health system, turning more directly against its medicalized nature.

„Peer support can make a good contribution to deconstructing the psychiatry system.” (MHW, interview, QUAL 1)

Such an expectation contrasts with experiences in which PSWs strongly rejected the position of criticizing mental healthcare systems:

“And I have also experienced peers who have said: ‘We can’t do anything with the criticism and with the system criticism that was originally inscribed in peer counselling. We just want to give good treatment and have no desire to actually change the whole psychiatric system.’” (MHW, interview, QUAL 1

On the other hand, the use of drug treatments was viewed as an essential component of mental health care by some MHWs, a perspective that could stand in opposition to those of PSWs who are critical of medication. Such disagreement was exemplified by one participant, who stated that a PSW was not hired because of her opposition to psychiatric drugs.

“Because we once had a problem with a peer, and it wasn’t nice. She was someone who intervened categorically and spoke negatively about the medication. That does not fit into a hospital like ours”. (MHW, focus group, QUAL 2)

Such conflicts also arose when the positions of MHWs and PSWs diverged regarding the effect of PSW input on service users, because in some cases, MHWs considered the impact of the PSW was stressful:

“It was the case that this peer worker sat in the day room with the patients and only railed against any medication, so to speak. It drove the patients crazy and was intense. So that was the tenor, that was the problem. If someone sits there in a differentiated way and says, it worked for me in such and such a way, that would not be a problem, that would rather be desirable to talk about one’s own experiences.” (MHW, interview, QUAL 1)

From the perspective of both groups, these and other conflicts could be more easily resolved if polarized/polarizing attitudes were avoided. The goal of both groups was to accommodate different perspectives in the team without tending to polarize.

## Discussion

The present study reveals that PSWs’ attitudes towards the medical model, as we have operationalized it (see 2.4.), are diverse. Some PSWs see psychiatric diagnoses as valuable for their own recovery and interactions with clients, whereas others view them as reductive and stigmatizing. Similarly, whereas all interviewed PSWs reported stepping back and referring service users to doctors during discussions of medication, some found psychiatric drugs beneficial, whereas others held more critical views. Such heterogeneity also applies to MHWs expectations: whereas some of them were disappointed that the PSWs’ attitudes were not critical enough, others perceived such criticism to be in conflict with their own attitudes and with what medical treatment should be. This is a challenge and can disturb PSW’s implementation in healthcare settings.

## Colliding Attitudes between MHWs’ Expectations and PSWs Positions and Attitudes

This tension is discussed in the literature. For instance, Moore and Zeeman (2020) describe conflicts between attitudes usually held by medical staff in favor of a biomedical model and those of PSWs, which tend to be more related to social models stressing the importance of supportive relationships. According to Moore and Zeeman, these conflicts are usually exacerbated by the time constraints of clinical practice, forcing the prioritization of one attitude over another. In this context, Byrne et al. (2016) introduced the concept of “worlds colliding” in their study as a metaphor for the frictions of the dominant medical model with the integration of PSWs’ and recovery approaches in mental healthcare facilities. Similarly, Potter (2021) argues that it is precisely the PSWs’ recovery orientation that clashes with the standard medicalized understanding of mental health care, becoming an obstacle to the implementation of PSW (Potter, 2021). However, in her classification of types of practices, Berard’s (2005) found mostly *compatible* attitudes between MHWs and PSWs, as the former as well as the latter supported recovery practices. Despite some resistance from professionals that are more attached to the medical model, both models seemed to ‘coexist with minimal conflict’ (Potter, 2021).

This heterogeneity resonates with our findings, but in a less clear-cut way: in our study, both the ascriptions to the MHWs and PSWs were less unified compared to the usual representations. For instance, many PSWs either participating in our study or represented by participants’ narratives seem to hold more ambivalent and, in some cases, even affirmative attitudes towards the medical model. Some of them reported finding psychiatric diagnoses to be helpful for understanding their crises, while others spoke in favor of medical treatment as a substantial part of their recovery. This affirmation may raise debates given the emancipatory origin of autonomous forms of peer support (Penney, 2018). Originally developed to combat or circumvent an overmedicalized mental healthcare system, PSWs within the boundaries of this system seem to have adapted some of these principles, a paradox that has also been described elsewhere using terminologies such as cooptation or appropriation (Russo & von Peter, 2022).

The same is true for MHWs: As shown by our results, not all of them positioned themselves as part of the medical model in a straightforward way. Some of the participating MHWs wanted PSWs to be part of their teams to promote more de-medicalizing attitudes. Others were astonished by PSWs’ adherence to diagnostic categories, hoping for more solidarity in relation to their own criticisms. In contrast, others reacted positively to PSWs’ affirmative position towards medicalized knowledge, at times making such attitudes a prerequisite for their employment. Thus, the conflicts between MHWs and PSWs in our study were neither definitive or generalizable. Instead, they emerged from complex situations in which both the attitudes of the PSWs and MHWs involved were more dynamic and diverse, a complexity and variety that has not been clearly stated in previous literature.

Accordingly, the terminology of “colliding attitudes” shall be employed in lieu of “colliding worlds” (Byrne et al., 2016) to convey the diverse positions and expectations held by both PSWs and MHWs. There are no clear-cut boundaries between these groups but rather a highly inter-individual heterogeneity of attitudes and position within each one – a heterogeneity that, in our opinion, may seriously impact the implementation of PSW. This risks creating insecurities on both sides, such that both MHWs and PSWs can never be certain how the person they encounter is positioned in relation to the medical model, or what his or her expectations towards this positioning are. These insecurities may be of less importance if there is sufficient time and resources to explain and negotiate these positions. However, they may be more problematic and even have material and existential consequences if they impact decisions about PSWs’ (non) employment.

## Colliding Attitudes Are Not Context-Less: The Role of Power Imbalances

The clashes of attitudes described above do not occur in power-free environments. Given the hierarchical nature of mental healthcare systems, MHWs hold more powerful positions that can influence organizational and patient-related decision-making at various levels (O’Shea et al., 2021). For example, in clinical contexts, PSWs are usually less able to effectively contribute their perspectives and attitudes due to the rhetorical dominance of clinical discourse (Vandewalle et al., 2016). Previous research supports the idea that MHWs hold power over PSWs, usually undergirded by the prevailing idea that “the expert knows best” (Byrne et al., 2016). This hierarchical culture, which reinforces the authoritative positions of professionals (Ehrlich et al., 2020), must be taken into account when considering the challenges faced by PSWs in relation to their attitudes towards the medical model.

The need for a new workforce in the field of mental health may present challenges to established professional groups, potentially impacting their power and status (Currie et al., 2012). We found that an important concern of some MHWs is their belief that PSWs may reject pure medical treatment approaches, for instance the use of medication. This concern has been interpreted in terms of a “defensive function”, as described by various authors (Cleary et al., 2011; Happell et al., 2015; Vandewalle et al., 2016), that could result in emotional distance or resentment towards PSWs. This emotional distance may serve as a protective mechanism for MHWs to preserve their beliefs and professional identity, as suggested by Hollway (2009). The hybrid position of PSWs, who are former users that work as part of mental health care teams, may be perceived as a threat to the division between “healthy professionals” and “sick service users.” Therefore, the defensive position of MHWs can be seen as maintaining the order of mental health care practices by renouncing more cross-border positions, ultimately affecting the implementation of PSWs. Although our data in this regard is limited, we have observed instances where some MHWs respond negatively to criticisms from PSWs. These reactions can be interpreted within the framework of this perspective.

Two situations were repeatedly reported in our data, revealing how these imbalances are negotiated on a day-to-day basis. Both situations related to questions about medication intake. First, the participating PSWs reported that they mostly stayed away from decision-making processes in relation to medication intake, which they understood to be the clinicians’ domain. Simultaneously, service users considered PSWs to play an important role in these questions and often start talking with them about medication. However, a critical attitude in relation to these questions on the side of the PSWs could be perceived as too radical by MHWs, as exemplified by the situation of the PSW that has not been employed for these reasons. Thus, PSWs seem to be trapped in situations in which they cannot autonomously engage with their own experiences and knowledge, regarding medication in particular. These examples surface some of the paradoxical attitudes held by MHWs: as long as they want to implement PSW, they want the PSWs’ attitudes to be aligned with their own. On the contrary, we saw an example of another MHW that pointed out that the function of PSWs in this regard is fundamental, since users have greater confidence in them. This opens up the possibility of a dialogue to better understand what experiences the patient has with psychotropic drugs. There is a risk of utilizing PSWs’ interventions to reinforce the desires of some MHWs to convince service users to take medication. These scenes imply that power imbalances ultimately determine the attitude of PSWs and their interaction with MHWs.

## Conclusions

According to our research, it appears that PSWs possess a greater diversity of attitudes towards key aspects of psychiatric treatment, such as medical diagnoses, than are typically acknowledged. This diversity may result in a clash in attitudes between PSWs and MHWs, particularly when these attitudes are polarized. This could negatively affect the implementation of PSW in established mental health services. Furthermore, it can impact the day-to-day practices of collaboration between both groups, leading to insecurities, false ascriptions, and deceitful expectations, thereby impeding close cooperation. The interactions between MHWs and PSWs can be challenging and complex. To improve implementation, it is crucial to depolarize the attitudes of both groups and foster a more inclusive work environment that integrates various perspectives. Less polarizing attitudes should not be understood here as uncritical acceptance of other positions. Rather, it means that various attitudes should not be presented in an exclusionary manner, whether directly or indirectly.

The attitudes and expectations of PSWs and MHWs may come together in a more *compatible* manner, as Potter (2021) found, if we recognize that both groups are not homogenous: they possess various perspectives, experiences, and positions. Such diversity should be considered before implementing PSW. Furthermore, preparation for implementing PSWs should take this diversity into account to adjust the expectations both PSWs and MHWs can place on PSW, and to enhance their capacity to adapt to a wider range of attitudes towards the mental health care system. A reflection on personal attitudes, objectives, and expectations (e.g., in the context of preparatory measures before implementation) could further facilitate the compatibility of attitudes between MHWs and PSWs and, thus, their successful implementation (Gillard et al., 2013; Moran et al., 2020).

## Limitations

Our study has some important limitations that we would like to acknowledge. One of these is the small sample size of the QUAL2 sub-study, which focused primarily on how MHWs perceive PSWs’ attitudes. Thus, in the QUAL2 phase, PSWs were not interviewed, which may have impeded a wider spectrum of perspectives on this issue. Another important limitation is the pragmatic operationalization of the medical model. Focusing on medical diagnoses and psychotropic drugs was necessary to organize the vast material of QUAL1. However, we consider this to be reductionism, as far as the medical model entails more aspects and elements.

## Supplementary Information

Below is the link to the electronic supplementary material.

Supplementary file1 (DOCX 19 KB)

Supplementary file2 (DOCX 17 KB)

Supplementary file3 (DOCX 26 KB)

## Abbreviations

MHWs: Mental health workers (plural)
MHW: Mental health worker (singular)
PSWs: Peer support workers
QUAL: Qualitative phase
QUAN: Quantitative phase
