# Supplementary material for: Attitudes of Peer Support Workers towards the Medical Model: A Qualitative Study from the Viewpoints of Peer Support Workers and Mental Health Staff
Source: Community Ment Health J. 2025 Feb 13;61(6):1138–47. doi: 10.1007/s10597-025-01454-z (PMC12228647; doi:10.1007/s10597-025-01454-z)
Supplement: Supplementary file 1 — Supplementary file1 (DOCX 19 KB) [file 10597_2025_1454_MOESM1_ESM.docx]

Supplementary material 1 — Sociodemographic data

Table 1 Demographic information QUAL1

| QUAL 1 | | |
| --- | --- | --- |
|  | PSWs | MHWs |
| Gender |  |  |
| w | 27 | 5 |
| m | 5 | 10 |
| other | 0 | 0 |
| Total | 33 | 19 |
|  |  |  |
| Age |  |  |
| < 20: | 0 | 0 |
| 20-30: | 0 | 2 |
| 31-40: | 9 | 4 |
| 41-50: | 8 | 4 |
| 51-60: | 13 | 7 |
| 61-70: | 3 | 2 |
| >71: | 0 | 0 |
| Total | 33 | 19 |
|  |  |  |
| Work as PSWs (years |  |  |
| >1 | 2 |  |
| 1-3 | 17 |  |
| 3-5 | 7 |  |
| 5-7 | 2 |  |
| 7-9 | 2 |  |
| > 9 | 3 |  |
| Total | 33 |  |
|  |  |  |
| How many years working with PSWs |  |  |
| 1-5 |  | 4 |
| 5-10 |  | 8 |
| > 10 |  | 3 |
| Total |  | 15 |
|  |  |  |
| Qualifications |  |  |
| EXIN | 28 |  |
| Other | 5 |  |
| Total | 33 |  |
|  |  |  |

Table 2 Demographic information QUAL2

| QUAL 2 | |
| --- | --- |
|  | MHWs |
| Gender |  |
| w | 6 |
| m | 2 |
| other | 0 |
| Total | 8 |
|  |  |
| Age |  |
| < 20: | 0 |
| 20-30: | 0 |
| 31-40: | 3 |
| 41-50: | 1 |
| 51-60: | 4 |
| 61-70: | 0 |
| >71: | 0 |
| Total | 8 |
|  |  |
| How many years working with PSWs |  |
| 1-5 | 2 |
| 5-10 | 3 |
| > 10 | 3 |
| Total | 8 |
