# Supplementary material for: Attitudes of Peer Support Workers towards the Medical Model: A Qualitative Study from the Viewpoints of Peer Support Workers and Mental Health Staff
Source: Community Ment Health J. 2025 Feb 13;61(6):1138–47. doi: 10.1007/s10597-025-01454-z (PMC12228647; doi:10.1007/s10597-025-01454-z)
Supplement: Supplementary file 2 — Supplementary file2 (DOCX 17 KB) [file 10597_2025_1454_MOESM2_ESM.docx]

**Expert Interviews Guide**

*(1^st^ level = main question; 2^nd^ level = further concretizing questions)*

1. What does the implementation of PSWs in psychiatry mean for MHWs?
   1. What attitude/attitude do the MHWs show at the beginning of the implementation?
   2. What worries/fears/insecurities arise?
   3. Are there different attitudes towards the implementation within MHWs?
   4. How does the attitude of MHWs change in the course of the cooperation?
   5. How does the behavior of the MHWs develop as a result of the presence of the PSWs?
2. What do PSWs contribute to the understanding of psychiatry?
3. What is the PSWs understanding of psychiatry and mental illness?
   1. To what extent do PSWs find psychiatric diagnoses useful? What do they mean for PSWs?
   2. What is the attitude of PSWs towards psychiatric drugs?
   3. Do the PSWs show different attitudes towards medication use?
   4. How do the differences in attitudes within the MHWs appear due to the use of PSWs?
   5. How does PSWs change MHWs’ attitudes towards psychiatric drugs?
   6. What role do PSWs play in MHWs’ intentions regarding medication use?
4. What is the attitude of the PSWs towards the medical model?
   1. What attitude towards diagnoses? How is the attitude of MHWs in this regard influenced by co-working with PB?
   2. Are there paradoxical attitudes of PSWs towards diagnoses?
   3. How important are/were the diagnoses for PSW in their crises?
   4. What does it mean for the understanding/concept of psychiatry to integrate the patient's perspective into treatment teams?
   5. How does this change the understanding of psychiatry and psychiatric disorders on a theoretical level?
   6. Do PSWs complement/expand psychiatry with their perspective?
   7. To what extent is this perspective necessary/essential for psychiatry?
   8. Is professional vision limited in psychiatry? What do PSWs contribute to this?
   9. Do PSWs promote a better understanding of mental health crises?
   10. Do the PSWs play a corrective role for MHWs?
   11. How do PSWs reinforce the normality of the crisis experience?
   12. Is there a change in psychiatry as a result of the use of PSWs?
   13. To what extent is the medical model related to the financing of the health care system?
   14. Is this connection a disadvantage for a critique of the medical model?
5. Do PSWs contribute to a paradigm change in mental health?
   1. Does the use of PSWs promote a different mentality of the MHWs?
   2. Are PSWs additional actors in a change in psychiatry that is already underway?
   3. Do PSWs promote a cultural change in psychiatry?
   4. Do PSWs require a cultural change in psychiatry?
   5. Is the image of psychiatry as a "repair shop" being deconstructed by PSWs?
   6. Is the thinking of the MHWs more biological?
   7. Is current psychiatry too technical?
   8. Is current psychiatry too drug-centered?
6. How do PSWs change the tendency of MHWs to think in terms of clinical diagnoses?
   1. Is it easier to perceive the person behind the diagnosis?
7. Will PSWs lead to a democratization of psychiatry?
   1. What role does the social/society play in the conception of psychiatry?
