# Supplementary material for: Attitudes of Peer Support Workers towards the Medical Model: A Qualitative Study from the Viewpoints of Peer Support Workers and Mental Health Staff
Source: Community Ment Health J. 2025 Feb 13;61(6):1138–47. doi: 10.1007/s10597-025-01454-z (PMC12228647; doi:10.1007/s10597-025-01454-z)
Supplement: Supplementary file 3 — Supplementary file3 (DOCX 26 KB) [file 10597_2025_1454_MOESM3_ESM.docx]

**Code System**

| **CODES** |
| --- |
| **1 Influence of Medical Model on psychiatric practices** |
| 1.1 MM determines power relations |
| 1.2.2 Problem of responsibility |
| 1.2.2.1 Some PSWs take responsibility |
| 1.2.2.2 Some PSWs relinquish responsibility |
| 1.2.3 Knowledge is power (power relations and roles) |
| 1.2.3.1 Violence/exercise of power by diagnoses and drugs |
| 1.2.3.2 MHWs are in a different position of power |
| 1.2.3.3 Professionals know that PSWs is subjective (epistemic injustice) |
| 1.2.4 Psychiatry separates healthy and sick people |
| 1.2.4.1 Biological model predominance vs. bio-psycho-social model |
| 1.2.4.2 Psychiatry as a repair shop |
| 1.2 Need for change |
| 1.2.1 Need for a critical/differentiated attitude |
| 1.2.2 Psychiatry must be more humane |
| 1.2.2.1 This would unsettle the system |
| 1.2.2.2 Classic roles need to be defused |
|  |
| **2 Significance of the diagnoses for PSWs and the togetherness with non-peer-staff** |
| 2.1 Criticism of diagnoses in psychiatry |
| 2.1.1 Critical attitude is independent of profession |
| 2.1.2 Psychiatry jargon leads to diagnoses |
| 2.1.3 Dx are (not) something solid |
| 2.2 Differing attitudes at the PSWs |
| 2.2.1 Reluctant attitude |
| 2.2.1.1 Psychiatry should focus less on diagnoses and more on the context of life |
| 2.2.1.2 Some reject diagnoses |
| 2.2.2 Open attitude |
| 2.2.2.1 This is seen as paradoxical by the MHWs |
| 2.2.2.1.1 Diagnoses-centered understanding on the part of PSWs |
| 2.2.2.1.2 Line is more critical of diagnosis than PSWs |
| 2.2.2.2 Diagnoses can be helpful |
| 2.2.2.2.1 Being able to name/comprehend/classify things |
| 2.2.2.2.2 Acceptance/dismissal of responsibility ("it can happen to anyone") |
| 2.2.2.2.3 Diagnoses help with acceptance |
| 2.2.2.2.4 (biological) explanation of one's own suffering |
| 2.2.2.3 Belonging/Location/Community Sense |
| 2.2.2.4 Diagnoses as something identity-giving |
| 2.2.3 PSWs don't care much about diagnoses |
|  |
| **3 Attitudes towards psychotropic drugs** |
| 3.1 Attitude of MHWs |
| 3.1.1 No critical attitude |
| 3.1.2 Critical attitude |
| 3.1.3 Differing opinions depending on the drug group |
| 3.1.4 Users should decide freely |
| 3.2 Attitude of the PSWs |
| 3.2.1 Reluctant attitude |
| 3.2.2. Role in working with users |
| 3.2.3. Role within the institution |
| 3.2.2 No intervention in this field |
| 3.2.2.1 PSWs refer to doctors |
| 3.2.2.2 Drugs are not their competence |
| 3.2.3 Open attitude |
| 3.2.3.1 PSWs must justify themselves |
| 3.2.3.2 Good experience with drugs |
| 3.3 Psychotropic drugs as a reductionist tool |
| 3.3.1 Medication is easier as other options |
| 3.3.2 Medications hide the real problem |
| 3.4 Need for psychotropic drugs |
| 3.4.1 Duty to provide information is usually not fulfilled |
| 3.4.2 Need for a critical attitude |
| 3.4.3 Polyphony |
|  |
| **4 Structural dependencies on diagnoses and psychotropic drugs** |
| 4.1 Diagnoses justifies coercive measures |
| 4.2 Financing of care |
| 4.2.1 Economically Oriented Care |
| 4.2.2 (No) freedom within the system |
| 4.2.3 Deficit-oriented understanding of disease/treatment |
|  |
| **5 MHWs‘ Attitude change** |
| 5.1 Undesirable developments of coworking with PSWs |
| 5.1.1 Unrealistic expectations of PSWs |
| 5.1.2 Trend of Participatory Psychiatry |
| 5.1.2.1 Danger of implementing PSWs without changing the institution |
| 5.1.3 PSWs feel gaps in the system |
| 5.1.3.1 Financing and structural problems (e.g. understaffing) |
| 5.1.4 Danger of adaptation of PSWs to the traditional structures |
| 5.1.4.1 Using PSWs for your own strategy |
| 5.1.4.2 Strengthening the balance of power |
| 5.1.4.3 Loss of critical attitude |
| 5.2 Postural change prior to implementation |
| 5.2.1 Some teams already had a pre-culture |
| 5.2.2 Necessity of preparing the MHWs |
| 5.2.3 Fears/insecurities |
| 5.2.3.1 Change comes/should come from above |
| 5.2.3.2 due to lack of preparation/participation (in the process) |
| 5.2.3.3 Shyness of one's own experience/concern |
| 5.2.3.4 Fear of PSWs posture |
| 5.3 Development by coworking with PSWs |
| 5.3.1 Change of attitude due to implementation |
| 5.3.1.1 More Understanding for users life contexts |
| 5.3.1.1.1 Replacing Distance with Proximity |
| 5.3.1.1.2 Criticism of the concept of compliance |
| 5.3.1.1.3 more critical handling of / less coercive measures |
| 5.3.1.1.4 MA learn through PB* the personal experience of NU |
| 5.3.1.1.5 Life contexts more important / Seeing people behind the diagnosis |
| 5.3.1.1.6 More attention to drug use (side effects, dosage...) |
| 5.3.1.2 Less separation between sick and healthy |
| 5.3.1.2.1 Separation MA/NU is no longer correct |
| 5.3.1.3 More critical to one’s own attitude |
| 5.3.1.3.1 Reinforcing the normality of the crisis experience |
| 5.3.1.3.2 More openness with regard to one's own concern |
| 5.3.1.3.3 Criticism of one's own role |
| 5.3.2 Less change as expected |
| 5.3.2.1 Still very much paid attention to diagnoses |
| 5.3.2.2 Some MHWs envy PSWs |
| 5.3.2.3 Fundamentals remain unchanged |
| 5.3.2.4 pessimistic outlook on the future |
| 5.3.2.5 Some MHWs block PSWs |
| 5.3.3 Positive development |
| 5.3.3.1 Other teams get to know PB*/want PB* too |
| 5.3.3.2 Employees want more PB* |
| 5.4 Changes in communication between actors |
| 5.4.1 Between NU and MA |
| 5.4.1.1 More shared decision making |
| 5.4.1.2 less technically speaking |
| 5.4.2 Within the team |
| 5.4.2.1 more respectful language about NU |
